# Hierarchically Structured Porous Electro-Conductive Aerogels for All-Solid-State Flexible Planar Supercapacitors with Cyclic Stability

**DOI:** 10.3390/gels12030221

**Published:** 2026-03-07

**Authors:** Huixiang Wang, Kaiquan Zhang, Ya Lu

**Affiliations:** 1 Department of Biological Sciences, Xinzhou Normal University, Xinzhou 034000, China; zhangkaiquan@xztu.edu.cn; 2 School of Automotive Engineering, Wuhu University, Wuhu 241008, China

**Keywords:** cellulose nanofibers, aerogels, supercapacitors, carbon nanotubes, manganese dioxide

## Abstract

Flexible supercapacitors have attracted significant attention as promising power sources for portable and wearable electronic devices. However, achieving simultaneous high power density, energy density and long-term cyclic stability in a simple device configuration remains a critical challenge. Herein, we report an all-solid-state flexible planar supercapacitor based on hierarchically structured cellulose nanofiber-carbon nanotube@manganese dioxide (CNF-CNT@MnO_2_) composite aerogels. The electrode architecture is rationally designed by first dispersing CNTs within a hydrophilic CNF scaffold to form a conductive three-dimensional network, followed by in situ oxidative polymerization of MnO_2_ onto the CNF-CNT fibrous skeleton. The hydrophilic CNFs network ensures thorough electrolyte penetration, the interconnected CNTs facilitate rapid electron transport, and the uniformly coated MnO_2_ layer provides substantial pseudocapacitance. The aerogel electrode with a low density of 14.6 mg cm^−3^ and a high specific surface area of 214.4 m^2^ g^−1^ delivers a specific capacitance of 273.0 F g^−1^ at 0.4 A g^−1^. The assembled planar supercapacitor, incorporating gel electrolyte and a flexible hydrogel substrate, achieves an impressive areal capacitance of 885.0 mF cm^−2^ at 2 mA cm^−2^, energy density of 122.9 μWh cm^−2^ and corresponding power density of 1000.0 μW cm^−2^. The device exhibits excellent electrochemical stability, retaining 83.3% capacitance after 2500 charge–discharge cycles, and outstanding mechanical flexibility, with 96.3% capacitance retention after 200 repeated bending cycles. Furthermore, multiple devices can be connected in series or parallel to proportionally increase output voltage or current, meeting the practical power requirements of electronic applications. This work offers a viable pathway toward high-performance, durable energy storage solutions for next-generation wearable electronics.

## 1. Introduction

The rapid proliferation of portable, wearable and flexible electronic devices has catalyzed an urgent demand for advanced energy storage systems that are not only compact and lightweight but also mechanically deformable, safe, and capable of delivering both high energy and power density [1,2,3]. Among various energy storage technologies, flexible supercapacitors have garnered immense attention due to their exceptional power density, rapid charge–discharge kinetics, long cycle life and good operational safety [4,5,6]. Unlike conventional batteries, supercapacitors store energy either electrostatically (electric double-layer capacitance, EDLC) or through fast surface redox reactions (pseudocapacitance), enabling them to bridge the performance gap between batteries and conventional capacitors [7,8]. However, achieving high energy density without sacrificing power output while maintaining electrochemical stability under repeated mechanical deformations remains a critical challenge for the practical application of flexible supercapacitors.

To enhance the energy density of supercapacitors, extensive research has focused on developing advanced electrode materials that combine high electrical conductivity with substantial charge storage capacity [9,10]. Carbon nanotubes (CNTs) represent one of the most promising EDLC materials due to their extraordinary electrical conductivity, high mechanical strength, large specific surface area and excellent chemical stability [11,12,13]. When incorporated into electrodes, CNTs form conductive percolation networks that facilitate rapid electron transport, thereby improving rate capability and power performance [14]. However, pristine CNT-based electrodes suffer from severe aggregation due to strong van der Waals forces, which reduces accessible surface area, limits electrolyte ion diffusion and ultimately compromises specific capacitance. Moreover, the purely electrostatic charge storage mechanism of CNTs restricts their energy density, making it challenging to meet the growing energy demands of modern electronics.

Pseudocapacitive materials, such as conducting polymer and transition metal oxides, have been extensively explored to introduce Faradaic charge storage, thereby significantly boosting the energy density of supercapacitors [15,16]. Among them, manganese dioxide (MnO_2_) stands out due to its high theoretical specific capacitance, natural abundance, low cost and environmental benignity [17,18]. MnO_2_ stores charge through reversible surface redox reactions, offering much higher charge storage capacity than carbon-based EDLC materials [19]. However, the practical application of MnO_2_ is hampered by its inherently low electrical conductivity and poor structural stability during repeated charge–discharge cycles, leading to sluggish reaction kinetics, limited rate capability and rapid capacitance fading [20]. Therefore, integrating MnO_2_ with highly conductive frameworks to form hybrid architectures is essential to enhance both electronic and ionic transport while mitigating mechanical degradation.

Cellulose nanofibers (CNFs), derived from abundant and renewable biomass resources, have emerged as attractive building blocks for flexible energy storage devices [21,22,23]. CNFs possess high mechanical strength, excellent flexibility, rich surface chemistry (with numerous hydroxyl and carboxyl groups), natural hydrophilicity, and the ability to form three-dimensional porous networks [24,25,26]. These characteristics make CNFs ideal structural scaffolds and dispersing agents for conductive nanomaterials like CNTs. The hydrophilic nature of CNFs promotes intimate contact with aqueous or gel electrolytes, facilitating ion penetration throughout the electrode, while their fibrous network can effectively buffer volume changes in active materials during cycling [27]. Furthermore, CNF-based hydrogels and aerogels can serve as flexible, lightweight and mechanically robust substrates for the fabrication of fully integrated, all-solid-state supercapacitors, eliminating the need for binders, separators or metal current collectors [28].

In this work, a hierarchically structured CNF-CNT@MnO_2_ composite aerogel was developed for all-solid-state flexible supercapacitors. Hydrophilic CNFs with high aspect ratio served as both a dispersing agent for CNTs and a flexible three-dimensional scaffold. Uniformly dispersed CNTs formed an interconnected conductive network that provided express electron transport pathways. The MnO_2_ layer, deposited via in situ oxidative polymerization, delivered high pseudocapacitance while protected from structural failure. This integrated configuration showed several distinct advantages over previously reported traditional physically mixed composites. First, the CNF-templated dispersion of CNTs prevented the agglomeration typically observed in CNT-only networks, ensuring maximum utilization of the conductive phase. Second, the hydrophilic CNFs promoted thorough electrolyte penetration throughout the aerogel, enabling intimate contact between the electrolyte and the electroactive MnO_2_ shell. Third, the hierarchical pore structure facilitated rapid electrochemical kinetics. Fourth, the flexible CNFs scaffold buffered the volume expansion and contraction of MnO_2_ during repeated charge–discharge cycles, enhancing long-term stability. The optimized CNF-CNT@MnO_2_ aerogel exhibited a low density, high specific surface area and a hierarchical porosity. The aerogel electrode delivered high specific capacitance with excellent rate capability and cycling stability. When assembled into an all-solid-state flexible planar supercapacitor, the device achieved a high areal capacitance, energy density and power density. Notably, the supercapacitor demonstrated both electrochemical and mechanical robustness. This work provided a viable strategy for developing high-performance, durable energy storage solutions for next-generation wearable electronics.

## 2. Results and Discussion

### 2.1. Chemical Structure Analysis

**Figure 1:**
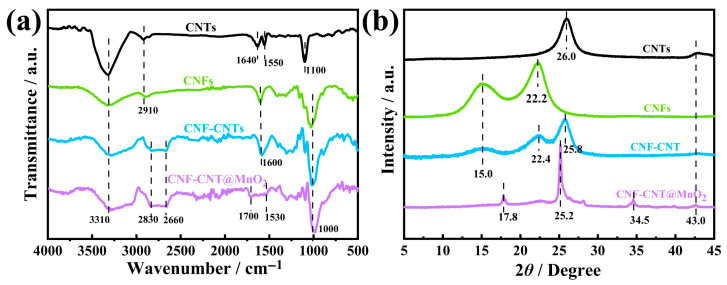
(**a**) FTIR and (**b**) XRD patterns of CNTs, CNFs, CNF-CNT and CNF-CNT@MnO_2_.

The FTIR spectra (Figure 1a) provided evidence for the successful integration of components and the presence of interfacial interactions. For all the samples, the peaks at 3310 cm^−1^, 2900 cm^−1^ and 1000 cm^−1^ corresponded to O-H, C-H and C-O-C vibrations [29,30]. For CNTs, the peaks at 1550 cm^−1^ and 1640 cm^−1^ were assigned to the carbon backbone vibrations of CNTs [31]. For CNFs, the peak at 1600 cm^−1^ was the characteristic peak of carboxyl groups [32]. The CNF-CNT nanohybrid mainly exhibited the typical absorption band of CNFs, indicating that CNTs were successfully carried and supported by CNFs as the bio-templates in the aqueous suspension. The characteristic peak of the C-H at 2900 cm^−1^ disappeared, and a broad peak was formed between 2830 cm^−1^ and 2660 cm^−1^, indicating the presence of interactions between CNFs and CNTs [33]. The interfacial interactions were critical for establishing a robust, electrically continuous network that could efficiently transport electrons to the MnO_2_ sites. After the incorporation of MnO_2_, the carboxyl peak at 1600 cm^−1^ broadened, suggesting the strong interactions between MnO_2_ and CNF-CNT [34]. Such interactions could anchor the MnO_2_ layer to the conductive scaffold, preventing detachment during repeated cycling, and promoted charge transfer across the interface, reducing internal resistance. The XRD patterns are shown in Figure 1b. Two diffraction peaks for CNTs at 2*θ* = 26.0° and 43.0° corresponded to the interlayer spacing (002) and the regular in-plane (100) diffraction (JCPDS No. 41-1487), respectively [35]. For CNFs, a prominent diffraction peak at 22.2° along with two weaker overlapping peaks at 2*θ* = 15.1° and 16.6° were indexed to (200), ($\overset{-}{11}0$) and (110) planes of cellulose I crystalline structure (JCPDS No. 50-2241) [36]. For CNF-CNT, the diffraction peak of CNTs at 2*θ* = 43.0° (100) disappeared, while the (002) interlayer diffraction peak of CNTs shifted to a lower angle, and the peak of CNFs at 2*θ* = 22.2° (200) shifted to a higher angle. These changes might result from the homogeneous dispersion of CNTs and the interactions between CNTs and CNFs [37]. The diffraction peaks of the MnO_2_ at 2*θ* = 17.8° (200), 25.2° (220), 34.5° (310), 40.0° (330) and 43.0° (301) matched with those of tetragonal α-MnO_2_ (JCPDS No. 44-0141) [38]. Compared with the XRD patterns of CNF-CNT, the diffraction peak of CNF-CNT@MnO_2_ at 2*θ* = 15.0° (200) disappeared, and the peak at 2*θ* = 26.0° (002) merged with the MnO_2_ peak at 2*θ* = 25.2° (220). These results confirmed the successful polymerization of MnO_2_ onto the CNF-CNT fibers, which could prevent the detachment of MnO_2_ layers during electrochemical charge–discharge cycles [39]. X-ray photoelectron spectroscopy (XPS) measurements were performed to provide direct evidence for the successful loading of MnO_2_ and the formation of interfacial interactions between MnO_2_ and the CNF-CNT skeleton (Figure S1). The CNF-CNT composite only exhibited characteristic peaks of C 1s (286 eV) and O 1s (533 eV), corresponding to the carbon backbones of CNFs and CNTs, and the oxygen-containing functional groups on the CNF surface [40]. For the CNF-CNT@MnO_2_, two distinct characteristic peaks of Mn 2p2/3 (642.3 eV) and Mn 2p1/2 (654.1 eV) and a weak Mn 3s (85 eV) peak were also observed. No other impurity elemental peaks were detected in the spectra, confirming the successful loading of MnO_2_ on the CNF-CNT fibrous skeleton via in situ oxidative polymerization [41], which was consistent with the XRD and FTIR results.

### 2.2. Morphology Analyses of the Aerogel Electrode

**Figure 2:**
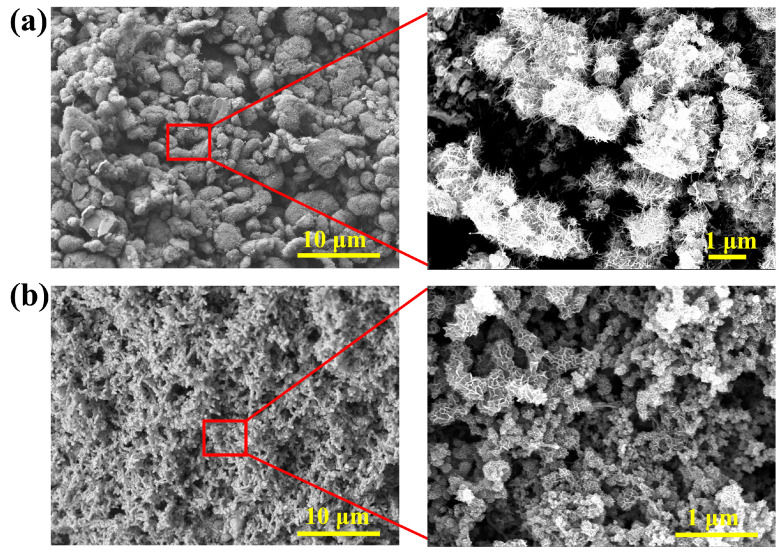
(**a**) SEM images of C@M and (**b**) C-C@M aerogels.

The microstructure of C@M and C-C@M aerogels observed by SEM is presented in Figure 2. The densities of the C@M and C-C@M were 205 mg cm^−3^ and 14.6 mg cm^−3^, respectively. The lightweight aerogel sample could be supported on a flower stamen (Figure S2). Due to van der Waals forces between CNTs, which caused mutual entanglement and prevented uniform dispersion, the CNT@MnO_2_ exhibited agglomerated bulk morphology (Figure 2a), with needle-like crystals grown on the surface. The SEM image of C-C@M revealed a fundamental morphological advantage conferred by the CNF scaffold (Figure 2b). Unlike C@M, the C-C@M aerogel exhibited a distinct three-dimensional fibrous network with uniform MnO_2_ coating (fiber diameter of 300 ± 100 nm), demonstrating that CNFs were essential for achieving homogeneous dispersion of CNTs and providing a template for controlled MnO_2_ nucleation. CNFs formed non-covalent interactions with CNTs to eliminate agglomeration and served as a growth substrate for the in situ oxidative deposition of MnO_2_. MnO_2_ was uniformly polymerized and coated on the outer surface of the fibrous network, forming a hierarchical porous structure. The pores between the fibers could act as transport channels for electrolyte ions, enabling deeper penetration of the electrolyte into the electrode material and facilitating interaction with more active material, thereby improving the electrochemical performance of the composite [42]. The internal CNT network facilitated efficient electron transport, enhancing both the electrical conductivity and electrochemical performance of the composite aerogel, while also serving as a supporting scaffold for MnO_2_ attachment. The MnO_2_ coating on individual fibers maximized the electrochemically active surface area while minimizing the ion diffusion distance within the solid phase. The needle-like MnO_2_ crystallites protruding from the fiber surfaces further increased surface roughness, creating additional active sites for pseudocapacitive reactions. This hierarchical architecture represented an optimized design for simultaneous high capacitance and fast kinetics. Compared to membranes prepared by high-temperature drying, aerogels fabricated via freeze-drying exhibited a more porous internal structure with a larger specific surface area, offering abundant ion-transport pathways and charge-adsorption sites, making them more suitable for electrode materials [43].

### 2.3. Specific Surface Area and Pore Structure of the Aerogel

**Figure 3:**
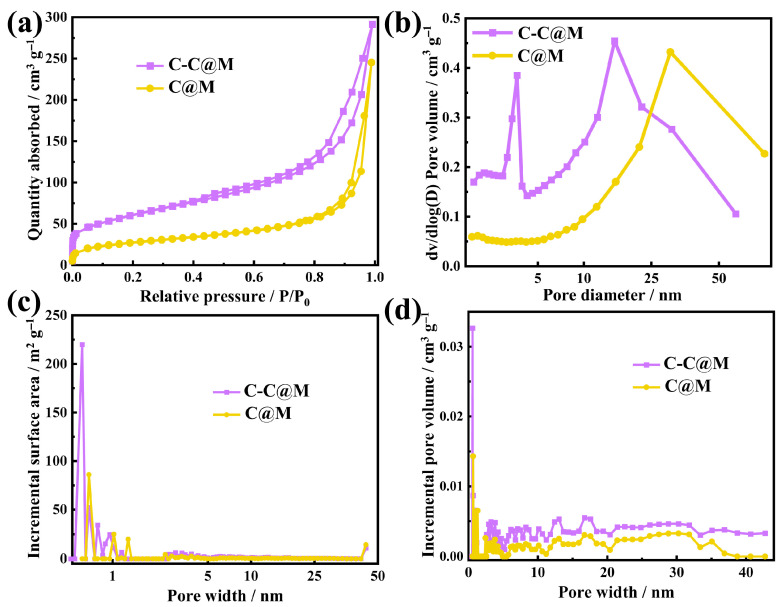
(**a**) Nitrogen adsorption/desorption isotherms and (**b**) micropore size distribution. (**c**) Curves of pore size versus specific surface area. (**d**) Curves of pore size versus pore volume.

The pores (micropores < 2 nm, mesopores 2–50 nm and macropores > 50 nm) of the composite materials could serve as channels for the entry and exit of electrolyte ions, facilitating the transmission and accumulation of charges, thereby enhancing the energy storage capability of the electrode materials [44]. As shown in Figure 3a, the adsorption capacity increased slowly within the range of P/P_0_ = 0.05–0.85. The change in adsorption capacity occurred within a relatively narrow pressure interval (0.8–1.0), indicating that the pore structure distribution in the aerogel membrane was uniform [34]. Upon the incorporation of CNFs, the specific surface area increased from 95.6 m^2^ g^−1^ (C@M) to 214.4 m^2^ g^−1^ (C-C@M). The relatively low specific surface area of C@M indicated severe agglomeration of CNTs, which not only reduced the accessible surface area but also blocked potential active sites within the aggregates. In contrast, the CNF-templated architecture exhibited more than a twofold increase in specific surface area, demonstrating that the fibrous network effectively prevented CNT entanglement and contributed additional surface area from the CNFs. The total pore volume of the C@M and C-C@M samples was 0.37 cm^3^ g^−1^ and 0.45 cm^3^ g^−1^, respectively, and the average pore diameters were 16.6 nm and 8.8 nm, respectively (Figure 3b). The aggregated CNT@MnO_2_ particles exhibited fewer pores and larger pore structures, which hindered the ion transmission and charge storage, resulting in a decrease in specific capacitance. On the contrary, the C-C@M aerogel had an average pore size of a mesoporous structure. The electrolyte ions could penetrate the interior of the aerogel structure through the interconnected mesopores. Notably, the C-C@M aerogel exhibited a distinct peak at ~3 nm in the pore size distribution, which arose from the CNF-templated uniform dispersion of CNTs and conformal MnO_2_ coating. In contrast, the agglomerated C@M aerogel lacked this concentrated peak, as severe CNT entanglement led to larger, irregular pores. The specific surface area of C-C@M was 370 m^2^ g^−1^ within the range of less than 2.0 nm, and 90 m^2^ g^−1^ within the range of 2.0–50 nm (Figure 3c). The specific surface area values of C@M (130 m^2^ g^−1^ within the range of less than 2.0 nm and 40 m^2^ g^−1^ within the range of 2.0–50 nm) were smaller than those of C-C@M. Based on the distribution curves of pore size and pore volume (Figure 3d), the C-C@M had the largest number of pores within a range of less than 2.0 nm, with a pore volume of 0.075 cm^3^ g^−1^, and the pore volume within the range of 2.0–50 nm was 0.2 cm^3^ g^−1^. The pore volumes of C@M within the ranges of less than 2.0 nm and 2.0–50 nm were 0.035 cm^3^ g^−1^ and 0.0885 cm^3^ g^−1^, respectively. This pore architecture had direct implications for the capacitive performance of the aerogel electrodes. The high specific surface area contributed by micropores (<2 nm) was primarily responsible for charge storage via the electric double-layer mechanism, as these pores provided abundant sites for ion adsorption at the electrode–electrolyte interface. However, the sole presence of micropores could lead to ion-transport limitations, especially at high current densities, due to restricted electrolyte accessibility. The well-developed mesopores (2–50 nm) served as efficient ion-diffusion channels that facilitated rapid electrolyte transport to the micropore interior. This hierarchical pore structure, which combined micropores for charge storage and mesopores for ion transport, was critical for achieving both high specific capacitance and rate capability. The higher micropore surface area of C-C@M directly contributed to the enhanced electric double-layer capacitance. Moreover, the larger mesopore volume and smaller average pore diameter of C-C@M ensured more efficient ion transport and greater accessibility of the electrolyte to the MnO_2_ pseudocapacitive sites. The interconnected mesopore network also accommodated the volume changes associated with repeated ion insertion/de-insertion during MnO_2_ redox reactions, contributing to the excellent cycling stability in the assembled supercapacitor. Thus, the optimized pore structure of the C-C@M aerogel was a key factor enabling the synergistic contribution of EDLC from CNTs and pseudocapacitance from MnO_2_, ultimately leading to enhanced overall electrochemical performance.

### 2.4. Electrochemical Performance of Aerogel Electrodes

**Figure 4:**
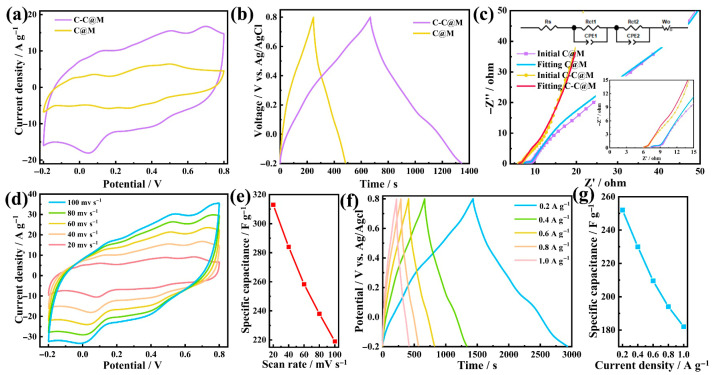
(**a**) CV curves at 40 mV s^−1^, (**b**) G-CD curves at 0.4 A g^−1^, and (**c**) EIS curves from C-C@M and C@M composite aerogel electrodes. Electrochemical characterization of C-C@M aerogel electrodes: (**d**) CV and (**e**) specific capacitances versus different scan rates, (**f**) G-CD and (**g**) specific capacitances versus different current densities.

To evaluate the electrochemical performance of the aerogel electrode, CV, G-CD and EIS tests were conducted. From the CV curves, two pairs of redox peaks of MnO_2_ could be observed (Figure 4a), indicating that MnO_2_ was successfully incorporated and participated in the electrochemical reaction. In C@M, the weak peaks suggested that MnO_2_ was not uniformly accessible. Agglomerated MnO_2_ particles experienced uneven current distribution. In C-C@M, the clear peaks indicated that the conductive CNT network effectively connected the MnO_2_ site to the electrode, enabling all MnO_2_ to participate simultaneously in the redox reactions. Furthermore, the larger integrated area under the CV curve of the C-C@M aerogel indicated a higher specific capacitance than that of the C@M aerogel. To elucidate the charge storage mechanism, b-value analysis and capacitive contribution quantification were performed. The b-values were 0.77 and 0.72, corresponding to anodic and cathodic peaks (Figure S4), respectively, confirming dominant surface-controlled capacitive behavior. According to Dunn’s method, the capacitive contribution was 75.3% at 20 mV s^−1^. This was because the MnO_2_ shell shortened ion diffusion paths, and the 3D conductive network accelerated electron transport, converting Faradaic reactions into rapid surface pseudocapacitance. Figure 4b presents the G-CD curves at a current density of 0.4 A g^−1^. The specific capacitance of C@M and C-C@M was 93.7 F g^−1^ and 273 F g^−1^, respectively. The C–C@M aerogel showed a smaller potential drop (IR drop) during the discharge process, suggesting lower charge-transfer resistance within the composite aerogel structure [45]. This could be attributed to the three-dimensional conductive network formed by uniformly dispersed CNTs, which enhanced overall electrical conductivity and effectively reduced the internal resistance [46]. According to the Nyquist plot derived from EIS (Figure 4c), the internal resistance (*R*_s_ = 6.0 Ω) and charge-transfer resistance (*R*_ct_ = 3.6 Ω) values of C-C@M were smaller than those of C@M (*R*_s_ = 7.2 Ω, *R*_ct_ = 5.3 Ω). The linear portion of the C-C@M aerogel in the low-frequency region was more parallel to the Y-axis than that of the C@M aerogel, indicating a higher specific capacitance. This result was consistent with the trends obtained from the CV and G-CD.

To further verify the electrochemical performance of the C-C@M aerogel electrode, CV at different scanning rates and GCD at different current densities were conducted. The CV curves exhibited nearly rectangular and symmetrical shapes, indicating efficient charge transfer within the electrode and good capacitive behavior [47]. As the scanning rate increased, the specific capacitance gradually decreased (Figure 4d), which was because the active MnO_2_ could not sufficiently participate in the pseudocapacitive reaction at high scanning rates. The specific capacitance values at scanning rates of 20, 40, 60, 80 and 100 mV s^−1^ were 312, 284, 258, 237 and 219 F g^−1^ (Figure 4e), respectively. The G-CD curve presented a symmetrical triangle shape (Figure 4f), which was mainly attributed to the electric double-layer energy storage behavior of CNTs within the aerogel. The capacitance values at current densities of 0.2, 0.4, 0.6, 0.8 and 1.0 A g^−1^ were 295, 273, 252, 237 and 225 F g^−1^ (Figure 4g), respectively. The decline in specific capacitance with increasing current density stemmed from the incomplete redox processes of the MnO_2_ and the insufficient time available for charge/discharge under high current density. The excellent electrochemical performance was attributed to the synergistic charge-storage mechanism of CNFs, CNTs and MnO_2_. The uniformly dispersed CNTs within the CNF scaffold formed a percolating conductive network and effectively reduced the electron transport distance, enabling MnO_2_ to participate in fast redox reactions even at high scan rates. The hydrophilic nature of CNFs, imparted by the abundant hydroxyl and carboxyl groups, ensured thorough electrolyte penetration into the aerogel interior. This was reflected in the high specific surface area and the hierarchical pore structure, which provided abundant electrochemically accessible sites. The CNF scaffold also acted as an effective dispersing agent for CNTs, preventing the agglomeration that would otherwise reduce the effective surface area and block ion transport pathways. Furthermore, the flexible CNF network buffered the volume changes in MnO_2_ during cycling, contributing to the excellent stability. The intimate contact between MnO_2_ and the CNF-CNT substrate was conductive to efficient charge transfer across the interface and prevented active material detachment.

### 2.5. Electrochemical Performance and Mechanical Stability of Supercapacitors

**Figure 5:**
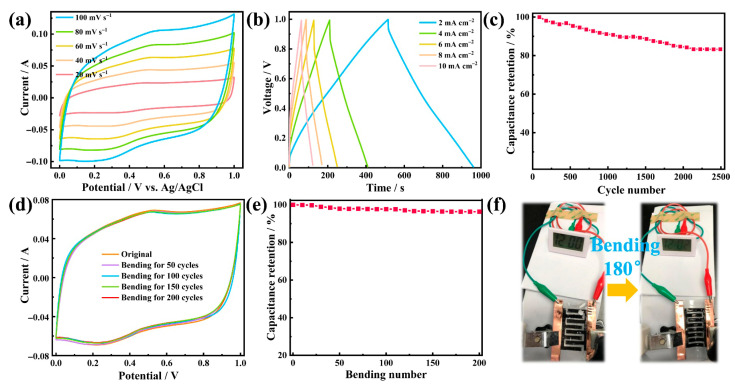
(**a**) CV curves obtained at 20, 40, 60, 80 and 100 mV s^−1^. (**b**) G-CD profiles tested at 2, 4, 6, 8 and 10 mA cm^−2^. (**c**) Cycling stability at a current density of 10 mA cm^−2^. (**d**) Variation in CV curves under repeated 180° bending. (**e**) Capacity retention versus number of bending cycles. (**f**) Photographs of capacitors powering an electronic watch in normal and bent states.

A planar all-solid-state integrated flexible supercapacitor was fabricated by assembling C-C@M aerogel electrodes and PVA/H_2_SO_4_ gel electrolyte with CNF/PVAB hydrogel matrix. The schematic diagram of the aerogel electrode is shown in Figure S3. CV measurement demonstrated that the advantages of the C-C@M electrode architecture were successfully translated to a fully assembled supercapacitor (Figure 5a). As the scan rate increased, the corresponding peak current increased, and the shape of the CV curves remained consistent, indicating stable charge–discharge performance. The presence of distinct redox peaks in the figure indicated typical pseudocapacitive behavior, which effectively enhanced the specific capacitance of the supercapacitor. The preservation of redox peaks at 100 mV s^−1^ confirmed that the pseudocapacitive contribution from MnO_2_ remained accessible even under fast charge conditions, attributable to the nanoscale MnO_2_ coating thickness and the efficient ion transport through the hierarchical pore network. The highly conductive CNTs significantly improved the conductivity by serving as express pathways for electron transfer. This facilitated efficient electron delivery to the MnO_2_ particles for the pseudocapacitive reactions, while simultaneously enabling rapid charge accumulation for electric double-layer capacitance. Meanwhile, CNFs formed a robust, three-dimensional skeletal framework. The inherent hydrophilicity promoted thorough electrolyte infiltration into the hierarchical porous network of the aerogel electrode, ensuring intimate contact with the electroactive materials (MnO_2_ and CNTs). This effectively increased the accessible area for electrolyte ion adsorption/desorption, thereby improving the overall electrochemical reaction efficiency.

The symmetrical triangular profile of the G-CD curves indicated excellent electrochemical reversibility and efficient charge transport within the capacitor (Figure 5b). The small observed IR drop further confirmed a low internal resistance. This favorable characteristic could be explained by the high ionic conductivity of the PVA/H_2_SO_4_ gel electrolyte and the planar device configuration, which collectively minimized ionic transport resistance. To evaluate the electrochemical stability of the capacitor, 2500 charge–discharge cycles were performed at a high current density of 10 mA cm^−2^ (Figure 5c). After long-term tests, the device retained 83.3% of its initial capacitance. The capacitance fading during long-term cycling was mainly attributed to the structural instability of MnO_2_ during redox reactions, the weakening of non-covalent interactions in the CNF-CNT conductive network, the dehydration and delamination at the electrode–electrolyte interface, and the minor dissolution of Mn ions in the acidic PVA/H_2_SO_4_ electrolyte. In future work, we aim to further enhance the durability through the following strategies: (i) MnO_2_ modification via heteroatom doping or covalent heterostructure construction to enhance structural stability and skeleton adhesion; (ii) CNFs chemical crosslinking and CNTs functional modification to strengthen the conductive framework and inhibit agglomeration; (iii) aerogel surface hydrophilic modification to improve interface compatibility and water retention; and (iv) ion-permeable protective layer coating on MnO_2_ to suppress Mn ion dissolution. To investigate the mechanical stability under bending deformation, the supercapacitor was bent by 180°. CV tests were conducted on the device after the 50th, 100th, 150th and 200th bends at a scan rate of 60 mV s^−1^ (Figure 5d). After repeated bending deformations, the electrochemical curve almost overlapped. The specific capacitance of the supercapacitor in its initial state was 858.0 mF cm^−2^ (Figure 5e). After 200 cycles of bending deformation, the capacitance remained at 826.2 mF cm^−2^ with a capacitance retention rate of 96.3%. Notably, even after 5000 cycles, the capacitance retention rate reached up to 86.7% (Figure S5), highlighting excellent mechanical durability. Figure 5f and Video S1 demonstrate the device powering an electronic watch in both flat and bent states, showcasing its potential application value as a flexible power source for wearable electronics. Compared with other similar flexible supercapacitors reported previously [48,49,50,51,52,53,54,55,56], the as-prepared CNF-CNT@MnO_2_ aerogel electrode-based supercapacitor presented excellent comprehensive performance (Table S1), especially the mechanical cyclic stability and high energy/power density, which was of great significance in flexible and portable electronics.

### 2.6. Series and Parallel Testing of Supercapacitors

**Figure 6:**
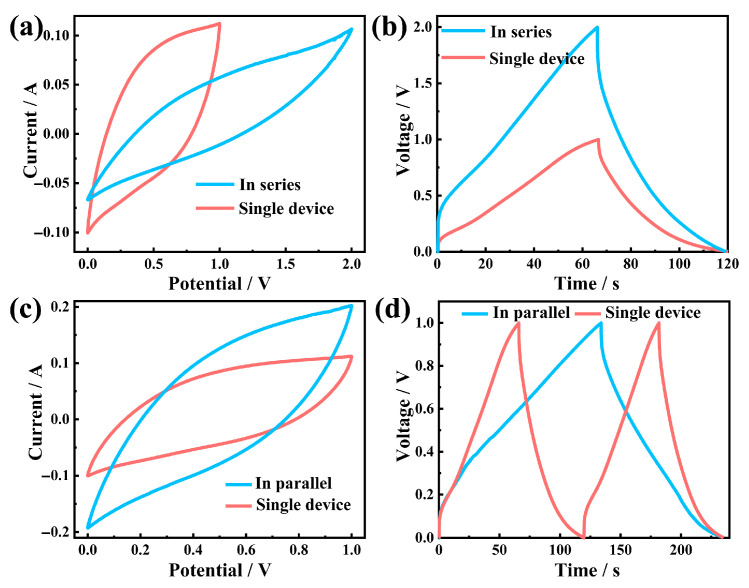
(**a**) CV and (**b**) G-CD curves of a single device and two devices in series. (**c**) CV and (**d**) G-CD curves of a single device and two devices in parallel.

To meet the power and energy requirements of microelectronic devices, several identical supercapacitors were connected in series or parallel. For a configuration consisting of two single-device series connections, the operating voltage window could be expanded to twice that of a single device, reaching a maximum of 2.0 V (Figure 6a). In the charge–discharge curve, the voltages of the two series-connected capacitors could also reach twice that of a single capacitor (Figure 6b). Compared with a single device, when operating within the same voltage range, the output current of the parallel devices increased by a factor of two (Figure 6c). Under the same charge and discharge current, the charge and discharge time were also twice that of a single device (Figure 6d), which proved that the capacitance had also increased to twice that of a single device. The as-prepared capacitors exhibited excellent characteristics of being able to generate maximum current and voltage in a linear proportion. Individual capacitors could be freely combined as needed to increase voltage and current, thereby meeting the application requirements in practical scenarios. The materials and fabrication processes employed in this work offered promising prospects for scalability and practical manufacturing. CNFs were derived from abundant renewable biomass using industrially scalable processes, CNTs were increasingly available at competitive costs, and MnO_2_ precursors were inexpensive commodity chemicals. The fabrication steps, including ultrasonication, freeze-drying and solution-based in situ polymerization, were all amenable to scale-up with appropriate engineering adaptations. While challenges such as ensuring large-area uniformity of MnO_2_ coating remained, the fundamental materials and processes demonstrated provided a viable foundation for developing commercially relevant flexible energy storage solutions.

## 3. Conclusions

In summary, a hierarchically structured CNF-CNT@MnO_2_ composite aerogel was fabricated by employing CNFs as a support for CNTs, followed by the in situ oxidative formation of MnO_2_ on the resulting fibrous skeleton. CNFs with a high aspect ratio and abundant hydroxyl and carboxyl groups could uniformly disperse CNTs and serve as the skeletal framework for aerogels. The high electrical conductivity of CNTs and the high pseudocapacitance of MnO_2_ complemented each other, significantly enhancing the electrochemical performance. The abundant porous structure of the aerogel could serve as transport channels for electrolyte ions, and the increased contact area between the electrode and electrolyte resulted in a higher specific capacitance. The aerogel electrode exhibited a high specific surface area and low density, achieving a specific capacitance of 272.0 F g^−1^. The assembled all-solid-state flexible planar supercapacitor delivered an areal capacitance of 885.0 mF cm^−2^, with a maximum energy density of 122.9 μWh cm^−2^ and a corresponding power density of 1000.0 μW cm^−2^. After 200 bending deformations, the device retained 96.3% of its areal capacitance. This flexible supercapacitor with outstanding stability showed promising potential for applications in wearable electronic devices.

## 4. Materials and Methods

### 4.1. Materials

Bleached wood pulp was brought from Nippon Paper Chemicals CO., Tokyo, Japan. CNTs (>97%, diameter: 10–20 nm, length: 3–10 μm) were obtained from Shenzhen Nanotechnology Co., Ltd. (Shenzhen, China). Potassium permanganate (KMnO_4_), manganese acetate (Mn(CH_3_COO)_2_·4H_2_O), polyvinyl alcohol (PVA), sodium hypochlorite (NaClO), sodium tetraborate decahydrate (Na_2_B_4_O_7_·10H_2_O, >99.5%), sodium bromide (NaBr), 2,2,6,6-tetramethylpiperidine-1-oxyl (TEMPO, C_9_H_18_NO), hydrochloric acid (HCl) and sodium hydroxide (NaOH) were purchased by Shanghai Aladdin Biochemical Technology Co., Ltd. (Shanghai, China).

### 4.2. Preparation of CNFs

First, 0.033 g of TEMPO and 0.33 g of NaBr were added successively to 400 mL of deionized water, and stirred until completely dissolved. Then, 2 g of cellulose powder was added and thoroughly mixed. Next, a NaClO (15 mmol g^−1^ cellulose) solution was added to initiate the oxidation reaction. During the reaction process, 1 M NaOH solution was gradually added to adjust the pH value to 10.5. After 6 h, the obtained oxidized cellulose was filtered and washed with deionized water to reach neutrality. The oxidized cellulose was prepared into a 2 mg mL^−1^ suspension and subjected to repeated homogenization at a pressure of 700 Pa using a high-pressure homogenizer for 4 times. The concentration of the final nanocellulose was adjusted to 0.4 wt% for later use.

### 4.3. Preparation of CNF-CNT@MnO_2_ Aerogel

CNF-CNT nanocomposite was prepared by adding CNTs to a CNF suspension at a mass ratio of CNTs:CNFs = 1:1, followed by ultrasonication at 400 W for 20 min in an ice-water bath. The CNF-CNT aqueous suspension was then concentrated to 5 mg mL^−1^ and freeze-dried to form a CNF-CNT aerogel. Then, 0.2 g of CNF-CNT aerogel was immersed in 20 mL of 0.5 M Mn(CH_3_COO)_2_·4H_2_O aqueous solution for 30 min. Subsequently, 20 mL of 0.3 M KMnO_4_ solution was added dropwise, and the mixture was heated in a 90 °C water bath for 5 h. The resulting CNF-CNT@MnO_2_ aerogel was then removed, repeatedly rinsed with deionized water to remove unreacted ions, and freeze-dried to remove residual moisture. The prepared CNF-CNT@MnO_2_ aerogel was denoted as C-C@M. For comparison, an aerogel prepared by polymerizing MnO_2_ onto CNT powder was designated as C@M.

### 4.4. Fabrication of Flexible Supercapacitors

First, a CNF/PVA/sodium tetraborate decahydrate (CNF/PVAB) hydrogel substrate was prepared. In brief, 2 g of PVA was added to 250 g of 0.4 wt% CNF aqueous suspension and heated at 90 °C until the PVA completely dissolved. Subsequently, 0.5 g of borax was slowly introduced into the mixture under stirring and heating until dissolved. The mixture was further heated to evaporate water until a hydrogel formed, yielding a CNF/PVAB hydrogel. The prepared CNF/PVAB hydrogel was first molded into a square shape with a thickness of 1 mm to serve as the capacitor substrate. The above aerogel was compressed under a pressure of 10 MPa into an aerogel film with a thickness of 0.1 mm, which was then laser-etched into serrated electrodes measuring 20 × 4 mm^2^. The mass loading of active material was ~6 mg cm^−2^. The aerogel electrodes were immersed in a PVA/H_2_SO_4_ gel electrolyte for 10 min. After removal, aerogel electrodes were placed on the top surface of the CNF/PVAB hydrogel substrate and dried at room temperature to fabricate a planar flexible supercapacitor.

### 4.5. Characterization

The density (g cm^−3^) of the aerogel was calculated based on the ratio of mass (g) to volume (cm^−3^). The microscopic structure and morphology of the aerogel were observed using a field-emission scanning electron microscope (FE-SEM, JSM-7600F, Nippon electronics Co., Ltd., Tokyo, Japan) with a working voltage of 15 kV. The chemical structure of the aerogel was characterized by Fourier transform infrared spectroscopy (FTIR, Nicolet iS50, Thermo Fisher Scientific Inc., Madison, WI, USA) using the ATR mode, with a wavelength range of 4000 to 500 cm^−1^. The crystal structure was characterized by X-ray diffraction (XRD, UItima IV, Rigaku, Tokyo, Japan), with the angle range of 2*θ* = 5–45° and the scanning rate of 5° min^−1^. X-ray photoelectron spectroscopy (XPS, AXIS-UltraDLD, Kratos Co., Manchester, UK) was used to analyze the elements of CNF-CNT and CNF-CNT@MnO_2_. The Brunauer–Emmett–Teller (BET) surface area and the Barrett–Joyner–Halenda (BJH) pore size distribution of the aerogel were characterized through nitrogen adsorption/desorption tests (ASAP 2020 HD88, Micromeritics Instrument Corporation, Norcross, GA, USA).

The electrochemical performance of the aerogel electrode was evaluated using a CHI760E electrochemical workstation (Shanghai Chenhua Instrument Co. Ltd., Shanghai, China) in a three-electrode configuration. The electrolyte employed was 1.0 M H_2_SO_4_. The working electrode was prepared by pressing composite aerogel (5 mg) onto nickel foam under a pressure of 10 MPa. A platinum sheet served as the counter electrode, and an Ag/AgCl electrode was used as the reference electrode. Electrochemical characterization was performed via cyclic voltammetry (CV), galvanostatic charge–discharge (G-CD), and electrochemical impedance spectroscopy (EIS). CV tests were conducted at a scan rate of 40 mV s^−1^ within a potential window of −0.2 to 0.8 V. G-CD measurements were carried out in the same potential range at a current density of 0.4 A g^−1^. EIS was performed under open-circuit conditions with an amplitude of 5 mV over a frequency range from 0.01 Hz to 100 kHz. The specific capacitance of the electrode was calculated from the G-CD curves using the following equation [57]:

(1) $$C=\frac{I\times ∆t}{m\times ∆U}$$

where *C* was the specific capacitance (F g^−1^), I denoted the discharge current (A), Δ*t* represented the discharge time (s), Δ*U* was the discharge voltage change after excluding the IR drop (V), and *m* referred to the mass of active material in the electrode (g).

The electrochemical performance of the supercapacitor was evaluated using a CHI760E electrochemical workstation under a two-electrode configuration at room temperature. CV measurements were performed at scan rates of 20, 40, 60, 80, and 100 mV s^−1^ over a voltage range of 0–1 V. G-CD tests were conducted at current densities of 2.0, 4.0, 6.0, 8.0, and 10.0 mA cm^−2^ within the same voltage window (0–1 V). Cycling stability was assessed by repeating the charge–discharge process for 2500 cycles at a current density of 10.0 mA cm^−2^. The flexibility and mechanical stability of the supercapacitor were evaluated by monitoring the capacitance retention under repeated bending deformations. The areal specific capacitance and energy density of the supercapacitor device were calculated using the following equations [58]:

(2) $$C_{a}=\frac{2I\times ∆t}{S\times ∆U}$$

(3) $$E_{a}=\frac{C_{a}\times ∆U^{2}}{2\times 3600}$$

(4) $$P_{a}=\frac{E_{a}\times 3600}{∆t}$$

where *C*_*a*_ was the areal specific capacitance (F cm^−2^), *I* was the discharge current (A), Δ*t* was the discharge time (s), Δ*U* was the voltage change range (V), *S* was the total area of the two electrodes (cm^2^), *E*_*a*_ was the areal energy density (Wh cm^−2^), and *P*_*a*_ was the areal power density (W cm^−2^).

The *b*-value was determined by the power-law relationship between the current (*I*) and scan rate (*v*). The charge-storage mechanism was analyzed through the following formula [59]:

(5) $$I=av^{b}$$

(6) log(I)=blog(v)+log(a)

where *a* was a constant, *b* was the slope of the logi-logv plot. *b* ≈ 1.0: Dominated by surface-controlled capacitive behavior. *b* ≈ 0.5: Dominated by diffusion-controlled behavior.

Dunn’s method deconvoluted the total current (i(V)) into capacitive current (*k*_1_*v*) and diffusion-controlled current (*k*_2_*v*^1/2^) via the following formula:

(7) $$i(V)=k_{1}v+k_{2}v^{1/2}$$

where *k*_1_ and *k*_2_ represented constants.

## Data Availability

The data supporting the findings of this study are available from the corresponding authors upon reasonable request.

## Supplementary Materials

The following supporting information can be downloaded at: https://www.mdpi.com/article/10.3390/gels12030221/s1, Figure S1: XPS spectra of CNF-CNT and CNF-CNT@MnO_2_; Figure S2: Photograph of CNF-CNT@MnO_2_ aerogel supported on a flower stamen; Figure S3: Schematic diagram of the aerogel electrode; Figure S4: log *I*−log ʋ curves of electrode. Figure S5: CV curves and capacitance retention rate after 5000 cycles of bending deformation. Table S1: Comparison of comprehensive performance with other recently reported flexible supercapacitors. Video S1: Supercapacitor powering an electronic watch during the bending process.
