# Supplementary material for: Hierarchically Structured Porous Electro-Conductive Aerogels for All-Solid-State Flexible Planar Supercapacitors with Cyclic Stability
Source: Gels. 2026 Mar 7;12(3):221. doi: 10.3390/gels12030221 (PMC13025631; doi:10.3390/gels12030221)
Supplement: Supplementary file 1 [file gels-12-00221-s001.zip › Supporting information.pdf]

Supporting information

Hierarchically structured porous electro-conductive aerogels for all-solid-state flexible planar supercapacitors with cyclic stability

Huixiang Wang,[a,*] *Kaiquan Zhang,*[a] *and Ya Lu*[b,*]

[a] Department of Biological Sciences, Xinzhou Normal University, Xinzhou, Shanxi 034000, China

[b] School of Automotive Engineering, Wuhu University, Wuhu, 241008, China

*** Corresponding authors:**

whx9111@163.com (H. Wang)

luyajiangsu@163.com (Y. Lu)

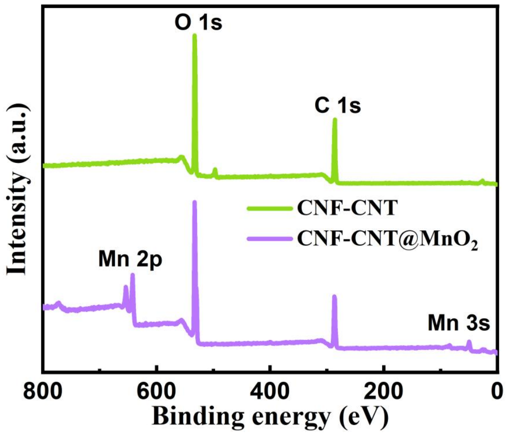

Figure S1. XPS spectra of CNF-CNT and CNF-CNT@MnO_2.

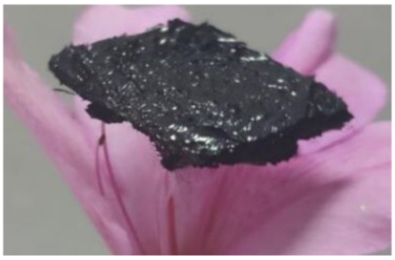

Figure S2. Photograph of CNF-CNT@MnO_2 aerogel supported on a flower stamen.

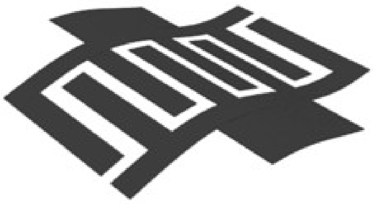

Figure S3. Schematic diagram of the aerogel electrode.

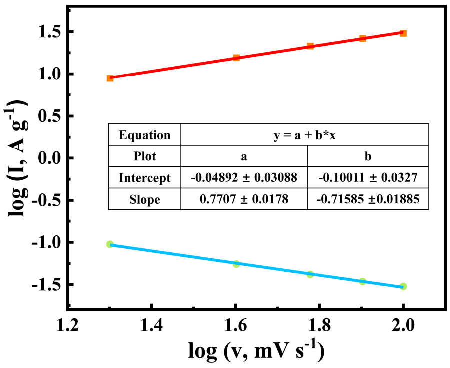

Figure S4. log I−log υ curves of electrode.

.

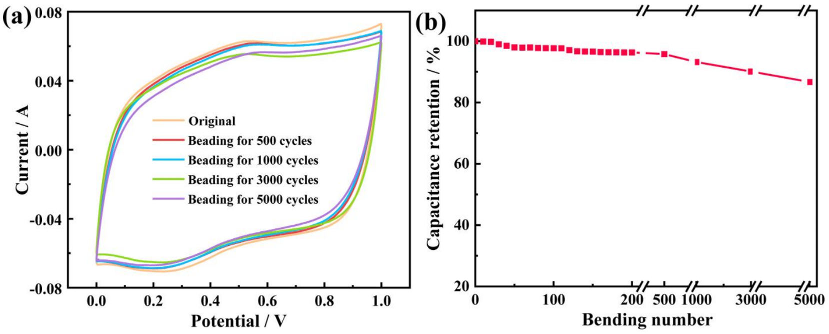

Figure S5. CV curves and capacitance retention rate after 5000 cycles of bending deformation.

Table S1. Comparison of comprehensive performance with other recently reported flexible supercapacitors.

Electrode	Areal capacitance	Energy density	Power density	Flexibility	Bending stability	Ref.
CNF-CNT@ MnO_2	858 mF cm^{-2} at 2 mA cm^{-2}	122.9 μW h cm^{-2}	1000 μW cm^{-2}	180° bending	86.7% after 5000 cycles	This work
Ni@MnO_2	10.75 mF cm^{-2} at 0.02 V s^{-1}	0.89 μWh cm^{-2}	0.88 mW cm^{-2}	radiuses of 10 mm	-	[1]
MnO_2/CNT	947 mF cm^{-2} at 1 mA cm^{-2}	0.4 μWh cm^{-2}	1.68 mW cm^{-2}	180° bending	80% after 100 cycles	[2]
TOCNFs/CNT/PANI	969 mF·cm^{-2} 0.5 mA·cm^{-2}	86.1 μWh·cm^{-2}	200 μW·cm^{-2}	-	-	[3]
PEDOT:PSS/ CNF	854.4 mF cm^{-2} at 5 mV s^{-1}	30.86 μWh cm^{-2}	0.22 mW cm^{-2}	180° bending	-	[4]
CNF/MXene/GI	188.2 mF cm^{-2} at 5 mA cm^{-2}	106.7 μWh·cm^{-2}	420 μWh cm^{-2}	Yes	-	[5]
PANI: PSS/CNP	460 mF cm^{-2} at 0.25 mA cm^{-2}	40.9 μWh cm^{-2}	100.5 μW cm^{-2}	180° bending	-	[6]
PDMS/CNTs/ PANI	408 mF cm^{-2} at 1 mA cm^{-2}	20 μW h cm^{-2}	100 μW cm^{-2}	120° bending	-	[7]
$Ti_3C_2T_x$-PANI@ CNTs	78.2 mF cm^{-2} at 0.1 mA cm^{-2}	2.02 μWh cm^{-2}	500 μW cm^{-2}	180° bending	81.1% after 10000 cycles	[8]
$Ti_3C_2T_x$/CNT/PC	212 mF cm^{-2} at 0.1 mA cm^{-2}	10.5 μW h cm^{-2}	29.8 μW cm^{-2}	Yes	-	[9]

References

1. Chen, Y.; Xie, S.; Li, G.; Jia, S.; Gao, X.; Li, X. 3D nanotube-structured Ni@MnO2 electrodes: Toward enhanced areal capacitance of planar supercapacitors. *Appl. Surf. Sci.* **2019**, *494*, 29-36.
2. Zhou, Y.; Cheng, X.; Tynan, B.; Sha, Z.; Huang, F.; Islam, M. S.; Zhang, J.; Rider, A. N.; Dai, L.; Chu, D. High-performance hierarchical MnO2/CNT electrode for

multifunctional supercapacitors. *Carbon* **2021**, *184*, 504-513.
3. Liu, S.; Chen, Y.; Dorsel, P.-K. P.; Wu, C. Facile preparation of nanocellulose/multi-walled carbon nanotube/polyaniline composite aerogel electrodes with high area-specific capacitance for supercapacitors. *Int. J. Biol. Macromol.* **2023**, *238*, 124158.
4. Du, H.; Zhang, M.; Liu, K.; Parit, M.; Jiang, Z.; Zhang, X.; Li, B.; Si, C. Conductive PEDOT: PSS/cellulose nanofibril paper electrodes for flexible supercapacitors with superior areal capacitance and cycling stability. *Chem. Eng. J.* **2022**, *428*, 131994.
5. Yuan, T.; Zhang, Z.; Liu, Q.; Liu, X.-T.; Tao, S.-Q.; Yao, C.-l. Cellulose nanofiber/MXene (Ti3C2Tx)/liquid metal film as a highly performance and flexible electrode material for supercapacitors. *Int. J. Biol. Macromol.* **2024**, *262*, 130119.
6. Liang, Y.; Wei, Z.; Wang, H.-E.; Flores, M.; Wang, R.; Zhang, X. Flexible and freestanding PANI: PSS/CNF nanopaper electrodes with enhanced electrochemical performance for supercapacitors. *J. Power Sources* **2022**, *548*, 232071.
7. Balboni, R. D. C.; Maron, G. K.; Masteghin, M. G.; Tas, M. O.; Rodrigues, L. S.; Gehrke, V.; Alano, J. H.; Andreazza, R.; Carreño, N. L. V.; Silva, S. R. P. An easy to assemble PDMS/CNTs/PANI flexible supercapacitor with high energy-to-power density. *Nanoscale* **2022**, *14* (6), 2266-2276.
8. Wang, Q.; Fang, Y.; Cao, M. Tailoring surface capacitance of Ti3C2Tx-PANI@CNTs nanoarchitecture for tunable energy storage and high-performance micro-supercapacitor. *Ceram. Int.* **2022**, *48* (15), 21935-21944.
9. Yang, K.; Luo, M.; Zhang, D.; Liu, C.; Li, Z.; Wang, L.; Chen, W.; Zhou, X. Ti3C2T /carbon nanotube/porous carbon film for flexible supercapacitor. *Chem. Eng. J.* **2022**, *427*, 132002.
